# Does hyperprolactinemia treatment affect pregnancy and perinatal outcomes?

**DOI:** 10.1590/1806-9282.20240634

**Published:** 2024-11-11

**Authors:** Amanda Carvalho Santos, Daniela Angerame Yela, Renan Massao Nakamura, Beatriz Cipriano Ribas, Pedro Henrique Rosa e Silva, Bianaca Mota, Cristina Laguna Benetti-Pinto

**Affiliations:** 1 University of Campinas, School of Medicine – Campinas (SP), Brazil.; 2 University of Campinas, School of Medical Sciences, Department of Obstetrics and Gynecology – Campinas (SP), Brazil.

**Keywords:** Hyperprolactinemia, Cabergoline, Bromocriptine, Obstetric deliveries, Perinatal care

## Abstract

**OBJECTIVE:**

The aim of this study was to assess obstetric and perinatal outcomes in women with hyperprolactinemia according to the type of treatment indicated, with cabergoline or bromocriptine.

**METHODS:**

A retrospective cohort study with 464 women diagnosed with hyperprolactinemia was undertaken at the endocrine gynecology outpatient clinic of a tertiary hospital from May 2002 to February 2022. All women diagnosed with hyperprolactinemia who were being treated with dopamine agonists (cabergoline or bromocriptine) and who became pregnant during the follow-up were included. Women whose medical records did not provide data related to pregnancy and delivery were excluded. The women were divided into two groups according to the type of treatment: bromocriptine or cabergoline. Clinical and laboratory characteristics and obstetric and perinatal outcomes, such as complications during pregnancy, type of delivery, and intrapartum complications, were evaluated in both groups.

**RESULTS:**

Of the 464 women evaluated, 72 became pregnant during the follow-up, 66 of them were using dopamine agonists, while 6 were not using medication. The mean age of the women was 28.3±6.8 years. Among the causes of hyperprolactinemia, 48.6% were idiopathic, 45.7% were tumoral, and 3.7% had other causes. Most women with idiopathic hyperprolactinemia used bromocriptine, while those with tumoral hyperprolactinemia used cabergoline (p=0.04). There was no difference in obstetric outcomes according to the type of treatment used. The majority of women did not have any complications during pregnancy (76.3%) or intrapartum (86.8%).

**CONCLUSION:**

Regardless of the type of previous drug treatment with dopamine agonists, hyperprolactinemia does not alter obstetric outcomes.

## INTRODUCTION

Hyperprolactinemia is defined by elevated levels of prolactin (PRL) in serum over the reference values defined by the laboratory^1^. The association with primary and secondary hypogonadism can lead to menstrual irregularities, amenorrhea, galactorrhea, infertility, decreased libido, and dyspareunia^2^.

When a woman looks for an assessment of infertility, one of the exams used to investigate the cause is prolactin levels. When hyperprolactinemia is found, the correct management can reverse infertility^3^. Treatment with dopamine agonist drugs aims to normalize prolactin levels and, thereby, reverse hypogonadism^4^.

The most commonly used medications in hyperprolactinemia treatment are bromocriptine and cabergoline, both dopamine agonist drugs^5^. Bromocriptine was the first drug commercialized and, due to its safety, was the drug of choice for women with reproductive desire, as it has a short exposure period due to its short half-life and would not cause any increase in unfavorable gestational and perinatal outcomes^6^. Cabergoline is considered the gold standard drug for treatment nowadays. During pregnancy, it is considered category B. Due to its long half-life, this drug raises concern about the risk of prolonged fetal exposure, even after the medication is withdrawn following the confirmation of pregnancy^4^.

There are some studies that evaluate the use of cabergoline for the treatment of hyperprolactinemia and its repercussions on prenatal and postnatal outcomes. Thus, the present study aims to evaluate the obstetric outcomes of women who became pregnant during hyperprolactinemia treatment according to the type of medication used.

## METHODS

This was a retrospective cohort study on women with hyperprolactinemia at the endocrine gynecology outpatient clinic of a tertiary hospital in the last 20 years. The clinic has a spreadsheet with syndromic and etiological diagnoses tabulated, and from these data, we selected the medical records of women followed for hyperprolactinemia. The medical records of women seen at the clinic from May 2002 to February 2022 were analyzed, totaling 499 medical records. In total, 35 records were excluded (women without a confirmed diagnosis of hyperprolactinemia or women who lost follow-up before starting treatment or making an etiological diagnosis of the cause of hyperprolactinemia).

The variables analyzed were age, etiology of hyperprolactinemia [idiopathic, tumoral—classified into microprolactinomas (size<10 mm) and macroprolactinomas (size ≥10 mm), drug, and others], diagnosis time (months), drug treatment used (bromocriptine or cabergoline), drug dose for treatment (mg/week with the bromocriptine tablet being 2.5 mg and the cabergoline tablet being 0.5 mg), treatment time (months), and serum prolactin (PRL—evaluated by electrochemiluminescence method, expressed in ng/mL whose normal value is less than 25 ng/mL). Blood collection was performed in our laboratory following standardization for stress-free sampling to reduce false positives of hyperprolactinemia.

For women who became pregnant, gestational outcomes were evaluated like complications during prenatal care (antenatal complications), obstetric outcomes (abortion, vaginal delivery, or cesarean delivery), fetal weight (adequate or small or big for gestational age), Apgar score, gestational age at birth (preterm, term, or post-term birth), birth weight, and delivery complications.

All pregnancies were spontaneous, and none of the women with tumors required surgical treatment.

This study was approved by the institution's Ethics and Research Committee (number: 46527721.2.0000.5404).

### Statistical analysis

A descriptive analysis of the sample was performed with absolute frequency values (n) and percentages (%), and descriptive statistics of the numerical variables, with mean and standard deviation values. Fisher's exact test was used to compare categorical variables, and the Mann-Whitney test was used to compare numerical variables. The significance level adopted for the statistical tests was 5%, that is, p<0.05.

## RESULTS

The study included 464 women with hyperprolactinemia, out of whom 72 became pregnant, representing 13.9% of the women during the follow-up period. Out of the pregnant women, 66 were using dopaminergic agonists (36 bromocriptine and 30 cabergoline), while 6 were not taking medication (they suspended the medication to try to get pregnant). The mean age of the women was 28.3±6.8 years, with 6.9% of them being younger than 20 years, 50.0% between 20 and 29 years, 37.5% between 30 and 39 years, and 5.5% between 40 and 49 years. The majority of women received treatment (91.6%), 68.0% were nulliparous, and most of them were still under treatment when they discovered they were pregnant (58.3%). The time of diagnosis of hyperprolactinemia was 93.9±68.3 months. The most commonly used drug was bromocriptine (54.5%) throughout the follow-up, as well as at the time of the pregnancy diagnosis (52.3%). The causes of hyperprolactinemia included idiopathic (48.6%), tumoral (45.8%), pharmacological (1.4%), and other causes (4.2%) (Table 1). All women discontinued the use of dopaminergic agonists in early pregnancy. The time interval between treatment and pregnancy was 60.79±29.89 months.

**Table 1:** Clinical characteristics of women with hyperprolactinemia that got pregnant during follow-up (n=72).

Variable		Mean±SD	%
Age (years)		28.3±6.8	
	<20		6.9
	20–29		50.0
	30–39		37.5
	>39		5.6
Nulliparous			68.0
Etiology			
	Idiopathic		48.6
	Tumoral		45.8
	Drug		1.4
	Others		4.2
Treatment			
	Cabergoline		41.7
	Bromocriptine		50.0
	No treatment		8.3
Initial prolactin levels (ng/mL)		139.7±121.7	
Prolactin levels before pregnancy (ng/mL)		37.0±42.1	
Diagnostic time (months)		93.9±68.3	
Treatment time (months)		33.1±38.4	

SD: standard deviation.

The majority of women with idiopathic hyperprolactinemia used cabergoline (70.0%), while those with tumoral hyperprolactinemia used cabergoline (59.3%) (p=0.04). Of the women with tumoral hyperprolactinemia, 14 had macroadenoma (7 in the cabergoline-treated group and 7 in the bromocriptine-treated group). There was no difference in obstetric outcomes according to the type of treatment used. Among the known obstetric outcomes, 45.6% resulted in vaginal delivery, 35.0% in cesarean section, and 19.3% in abortion. Most newborns were at term (94.8%) and had an appropriate weight for gestational age (86.8%). The mean Apgar score at 1 min was 8.4 and at 5 min was 9.5. Most women did not experience any complications during prenatal care (76.3%) or delivery (86.8%). There were no cases of fetal malformation, regardless of the type of medication used (Table 2).

**Table 2:** Clinical characteristics and obstetric outcomes of women with hyperprolactinemia according to the treatment with dopamine agonist—cabergoline or bromocriptine (n=66).

Variable	Cabergoline n=30 Mean±SD/%	Bromocriptine n=36 Mean±SD/%	p
Age (years)	30.3±5.4	28.8±8.4	0.39*
Etiology			0.04**
Idiopathic	30.0	70.0	
Tumoral	59.3	40.7	
Others	50.0	50.0	
Drug dose (mg/week)	1.9±4.4	26.3±12.7	<0.001*
Antenatal complications	18.7	18.1	1.00**
Delivery complications	12.5	18.1	1.00**
Obstetric outcomes			0.73**
Abortion	15.7	12.5	
Vaginal delivery	42.1	56.2	
Cesarean delivery	42.1	31.2	
Fetal malformations	0	0	1.00**
Fetal weight			1.00**
Adequate	81.2	81.8	
Small for gestational age	12.5	9.0	
Big for gestational age	6.2	9.0	
Gestational age			1.00**
Term	93.7	90.9	
Preterm	6.2	9.0	
Birth weight (grams)	3126.3±549.6	3153.6±498.3	0.90*

SD: standard deviation

* Mann-Whitney test

** Fisher's test.

## DISCUSSION

This study observed that 13.9% of women became pregnant during the follow-up period, and the majority of them were receiving medication treatment at the time of pregnancy. The most frequent diagnosis was idiopathic hyperprolactinemia, followed by secondary pituitary tumors. Regarding the outcome and complications related to prenatal and delivery, there was no difference between women using cabergoline or bromocriptine.

According to literature data, the use of drugs and prolactinomas are considered the main non-physiological causes of hyperprolactinemia^7^. Of all etiologies, the most common cause is the use of drugs that in some way affect the hypothalamic dopaminergic system and/or its pituitary receptors—increasing the transcription of prolactin, which in turn antagonizes the dopamine receptor, causing its depletion, with inhibition of hypothalamic dopamine production, or dopamine or serotonin reuptake, among other forms^8,9^. Prolactin-producing tumors, on the other hand, are responsible for 20–30% of pathological hyperprolactinemia cases^1^. In contrast to what was presented in this study, we see idiopathic hyperprolactinemia as the main cause (48.6%), followed by pituitary tumors (45.8%).

The term idiopathic hyperprolactinemia is reserved for women without an identified cause. However, it is known that in many cases, a mild prolactinoma may be present, too small to be identified by conventional radiological techniques. In other cases, hyperprolactinemia is presumably caused by hypothalamic regulatory dysfunction^9^.

The therapeutic management of choice for women with hyperprolactinemia is the use of dopaminergic agonists^10^. The majority of women in this study (91.7%) received drug treatment, with the use of bromocriptine slightly higher (50.01%).

A number of studies involving 2,587 pregnancies and the use of bromocriptine found no elevated risk of miscarriage, multiple pregnancies, or the occurrence of congenital malformations. These findings lead to the conclusion that bromocriptine does not have a negative impact on pregnancy, either for the mother or the fetus^11,12^.

Studies suggest that dopamine is an important hormone for motor and cognitive development, and dopaminergic neurons appear early in fetal development (around 6–8 weeks gestational age). Bromocriptine is known to cross the placental barrier, which suggests that cabergoline also crosses the barrier^4^. Animal studies have demonstrated this same behavior for cabergoline^6^. However, as cabergoline has a longer half-life, fetal exposure to the drug would also be prolonged, precisely during a critical phase of organogenesis, which makes the use of cabergoline potentially concerning^4^. Despite being limited, there are reports of several cases of pregnancy during cabergoline use without evidence of increased rates of spontaneous abortion, preterm birth, or multiple gestations. Long-term postnatal follow-up also did not show physical or mental abnormalities in the development of exposed children, indicating that cabergoline also does not have a deleterious effect on fetal development^6^.

Few of the studies in the literature demonstrate the safety of cabergoline during pregnancy and its effects on fetal development. However, it is known that cabergoline is safe in childhood and adolescence, and there are several reports of pregnancies while on cabergoline treatment that progressed without complications^13,14^. Of the women analyzed using cabergoline when becoming pregnant in the present study, whose outcomes were known, 81.2% had adequate birth weight, 93.7% had full-term gestation, 87.5% did not present complications during delivery, and 81.2% did not have complications during prenatal care. In addition, there was no statistically significant difference between the two drugs when compared to bromocriptine.

A Brazilian study confirms previous safety results on maternal and fetal outcomes in pregnancies after the use of cabergoline; however, continuing cabergoline after confirmation of pregnancy was associated with a higher rate of miscarriage^15^.

One study showed that the use of dopaminergic agonists (cabergoline and bromocriptine) during the first 5 weeks of pregnancy is safe and is not associated with an increase in the number of spontaneous abortions, congenital malformations, or other adverse neonatal and gestational outcomes^16^.

An Indian study that evaluated 31 pregnancies of women with hyperprolactinemia of pituitary adenoma etiology treated with cabergoline showed that most women (80%) delivered at term and that there was no difference in the gestational results of women who maintained or discontinued use of dopaminergic agonists during pregnancy^17^.

A prospective study that followed 329 pregnancies of women treated with cabergoline suggests that fetal exposure to cabergoline during early pregnancy does not induce any increase in the risk of miscarriage or fetal malformation^18^.

The limitations of this study are that it is a retrospective evaluation, and many of the women who became pregnant during treatment were lost to follow-up after confirmation of pregnancy, making it impossible to know the obstetric outcome of these cases. In addition, it was not possible to evaluate the long-term neuropsychomotor development of children during childhood and adolescence to correlate with exposure to a particular dopaminergic agonist. Another limitation is the absence of a control group of healthy pregnant women. Despite these limitations, the standardized follow-up period within an outpatient clinic provides robust evidence about the data. For complementary evaluation, randomized clinical trials could provide evidence on the safety of cabergoline use during pregnancy.

## CONCLUSION

Hyperprolactinemia, regardless of the type of medication treatment with cabergoline or bromocriptine, did not affect obstetric outcomes in this sample.

## References

[1] Soto-Pedre E, Newey PJ, Bevan JS, Greig N, Leese GP (2017). The epidemiology of hyperprolactinaemia over 20 years in the Tayside region of Scotland: the Prolactin Epidemiology, Audit and Research Study (PROLEARS). Clin Endocrinol (Oxf).

[2] Bonert V (2020). Do nothing but observe microprolactinomas: when and how to replace sex hormones?. Pituitary.

[3] Ruiz-Velasco V, Tolis G (1984). Pregnancy in hyperprolactinemic women. Fertil Steril.

[4] Glezer A, Bronstein MD (2014). Prolactinomas, cabergoline, and pregnancy. Endocrine.

[5] Casanueva FF, Molitch ME, Schelechte JA, Abs R, Bonert V, Bronstein MD (2007). Guías de la Pituitary Society para el diagnóstico y tratamiento de los prolactinomas. Endocrinol Nutr.

[6] Molitch ME (2015). Endocrinology in pregnancy: management of the pregnant patient with a prolactinoma. Eur J Endocrinol.

[7] Vilar L, Freitas MC, Naves LA, Casulari LA, Azevedo M, Montenegro R (2008). Diagnosis and management of hyperprolactinemia: results of a Brazilian multicenter study with 1234 patients. J Endocrinol Invest.

[8] Vilar L, Fleseriu M, Bronstein MD (2014). Challenges and pitfalls in the diagnosis of hyperprolactinemia. Arq Bras Endocrinol Metabol.

[9] Molitch ME (2001). Disorders of prolactin secretion. Endocrinol Metab Clin North Am.

[10] Majumdar A, Mangal NS (2013). Hyperprolactinemia. J Hum Reprod Sci.

[11] Turkalj I, Braun P, Krupp P (1982). Surveillance of bromocriptine in pregnancy. JAMA.

[12] Krupp P, Monka C (1987). Bromocriptine in pregnancy: safety aspects. Klin Wochenschr.

[13] Stalldecker G, Mallea-Gil MS, Guitelman M, Alfieri A, Ballarino MC, Boero L (2010). Effects of cabergoline on pregnancy and embryo-fetal development: retrospective study on 103 pregnancies and a review of the literature. Pituitary.

[14] Glezer A, Bronstein MD (2020). Prolactinomas in pregnancy: considerations before conception and during pregnancy. Pituitary.

[15] Sant’ Anna BG, Musolino NRC, Gadelha MR, Marques C, Castro M, Elias PCL (2020). A Brazilian multicentre study evaluating pregnancies induced by cabergoline in patients harboring prolactinomas. Pituitary.

[16] O’Sullivan SM, Farrant MT, Ogilvie CM, Gunn AJ, Milsom SR (2020). An observational study of pregnancy and post-partum outcomes in women with prolactinoma treated with dopamine agonists. Aust N Z J Obstet Gynaecol.

[17] Laway BA, Baba MS, Bansiwal SK, Choh NA (2021). Prolactinoma outcome after pregnancy and lactation: a cohort study. Indian J Endocrinol Metab.

[18] Colao A, Abs R, Bárcena DG, Chanson P, Paulus W, Kleinberg DL (2008). Pregnancy outcomes following cabergoline treatment: extended results from a 12-year observational study. Clin Endocrinol (Oxf).

